# Molecular Genetics and Breeding for Nutrient Use Efficiency in Rice

**DOI:** 10.3390/ijms19061762

**Published:** 2018-06-14

**Authors:** Jauhar Ali, Zilhas Ahmed Jewel, Anumalla Mahender, Annamalai Anandan, Jose Hernandez, Zhikang Li

**Affiliations:** 1 Rice Breeding Platform, International Rice Research Institute (IRRI), Los Baños, Laguna 4031, Philippines; jeweluplb@gmail.com (Z.A.J.); m.anumalla@irri.org (A.M.); 2 ICAR-National Rice Research Institute, Cuttack, Odisha 753006, India; anandanau@yahoo.com; 3 Institute of Crop Science, College of Agriculture and Food Science, University of the Philippines Los Baños, Laguna 4031, Philippines; joehernandez56@gmail.com; 4 Institute of Crop Sciences, Chinese Academy of Agricultural Science, Beijing 100081, China; zhkli1953@126.com

**Keywords:** NPK fertilizers, agronomic traits, molecular markers, quantitative trait loci

## Abstract

In the coming decades, rice production needs to be carried out sustainably to keep the balance between profitability margins and essential resource input costs. Many fertilizers, such as N, depend primarily on fossil fuels, whereas P comes from rock phosphates. How long these reserves will last and sustain agriculture remains to be seen. Therefore, current agricultural food production under such conditions remains an enormous and colossal challenge. Researchers have been trying to identify nutrient use-efficient varieties over the past few decades with limited success. The concept of nutrient use efficiency is being revisited to understand the molecular genetic basis, while much of it is not entirely understood yet. However, significant achievements have recently been observed at the molecular level in nitrogen and phosphorus use efficiency. Breeding teams are trying to incorporate these valuable QTLs and genes into their rice breeding programs. In this review, we seek to identify the achievements and the progress made so far in the fields of genetics, molecular breeding and biotechnology, especially for nutrient use efficiency in rice.

## 1. Introduction

Global rice production increased by three-fold over the past three decades despite rice production constraints and rising input costs. Rice is a nutritionally important cereal crop and staple food of Asia. There is an urgent need for developing high-yielding, nutritious, resource use-efficient and multi-stress-tolerant rice varieties to keep up with the tremendous human population growth, especially in Asia, where rice remains the primary source of caloric intake. The yields of rice grain had seen remarkable improvement during the green revolution and post‒green revolution. This increase in yield was primarily achieved through high-input-responsive varieties requiring more chemical fertilizers and pesticides and under an ample supply of irrigation water. This kind of approach that predominated over the past three to four decades now stands exhausted amidst our hope to raise productivity per se sustainably. We are now finding that yields are fast approaching a theoretical limit set by the crop’s efficiency in harnessing applied inputs. In exploratory managed experimental plots, N fertilizer retrieval in a single year averaged 65% for maize, 57% for wheat and 46% for rice [1,2]. Alterations in the scale of farming operations and management practices such as tillage, seeding, weed and pest control, irrigation and harvesting usually resulted in on-farm variation (lower nutrient use efficiency) and did not accurately reflect the efficiencies obtained in the experimental plot. N recovery efficiency on average ranges from 20–30% for farmer-managed fields under rainfed conditions, from 30–55% under irrigated conditions [3,4] and rarely exceeds 50%.

Over the years, the rice varieties bred did not improve in nutrient absorption and were not developed to maximize nutrient absorption, but they have the capacity to use less than 50% of the applied nutrients. Breeding rice cultivars with improved nutrient use efficiency (NuUE) is becoming a prerequisite for lowering production costs. Such cultivars with NuUE protects the environment by reducing fertilizer application, decreasing the rate of nutrient application losses to ecosystems, decreasing input costs and improving rice yield with a guarantee for sustainability in agriculture while maintaining soil and ground water quality. On the other hand, improvement of NuUE is an essential prerequisite for expanding crop production into marginal lands with low nutrient availability. In light of high energy costs and progressively unpredictable resources, future agricultural systems with concern for improving yield productivity need to be more fruitful and efficient, especially considering fertilizer and irrigation water. In this context, the identification and development of rice varieties with superior grain yield under low input conditions have therefore become a high breeding priority [5]. Even though significant genotypic differences in nitrogen use efficiency exist in rice, genetic selection for this trait has not been carried out systematically [6,7,8]. This may be primarily because of the complexity involved in the overall phenotype and its evaluation and the non-availability of genetic tools to use. However, with the recent use of high-throughput single nucleotide polymorphism (SNP) markers with ease and high precision, this area of research needs improvement for better understanding [9,10,11].

Genetic and physiological traits often change with the interaction with environmental variables. Plants are efficient in the absorption and use of nutrients in controlled environments. Therefore, there is a need for a systematic breeding program to develop cultivars with high NuUE and water use efficiency (WUE) [12,13]. The traits involved, particularly nutrient absorption, transport, use and mobilization, should be identified to enhance NuUE and coupled with best management practices for sustainable agriculture.

Use of the wild species of *Oryza* and native landraces becomes imperative for exploiting the untapped reservoir of useful QTLs and genes, especially to broaden the genetic basis of rice and to enrich existing varieties [14,15]. Genetic selection and plant breeding techniques helped to develop rice varieties that are resistant to pests, diseases and adverse environmental conditions such as drought, submergence and salinity. However, for improving NuUE in rice crop, a proper genetic selection approach is necessary. Superior N-efficient genotypes are required as evidenced from the low recovery of N fertilizer, associated economic and environmental concerns and the lack of adoption of more efficient N management strategies [16,17]. Nitrogen use efficiency (NUE) mostly depends on interactions and the use of the nutrient in a proper way, water availability, light intensity, disease pressure and genotype, which could also be improved through appropriate genetic manipulation [6]. Plant ability to absorb and use nutrients under various environmental and ecological conditions is largely influenced by the genetic makeup and physiological components [12]. There are two major approaches to understand NuUE. First, the nutrient deficiency stress triggers a response of plants to it, which may lead to the identification of the processes affecting it. It would help us to understand how to sustain plants under low nutrient inputs. The second approach would be to exploit genetic variability (both natural and induced) through innovative molecular breeding schemes.

Molecular linkage genetic maps and quantitative trait locus (QTL) mapping technologies are helpful for estimating the number and position of the loci governing genetic variation using different types of segregating and fixed populations. Characterizing these loci to their map positions in the genome, as well as their phenotypic effects and epistatic interactions with other QTLs and loci [18,19,20,21] has enabled us to explore the genetic loci associated with complex traits such as drought, salinity, disease, NuUE and insect resistance in crop plants [18,22,23,24,25,26,27,28,29]. The rapid advancement in genome sequencing technologies and marker-aided breeding approaches has resulted in a change in breeding methods, providing new opportunities [5]. Association mapping is a method used to identify genes and QTLs underlying quantitatively inherited variation based on a diverse set of fixed lines. It allows the discovery of QTLs/genes using historical phenotypic data and eventually leads to identifying gene functions, under used alleles and allele combinations that can be useful for crop improvement [30,31]. Genome-wide association mapping depends on the strength of linkage disequilibrium (LD) across a diverse population besides identifying the relationships between markers and traits of agronomic and evolutionary interest [32,33].

**Figure 1:**
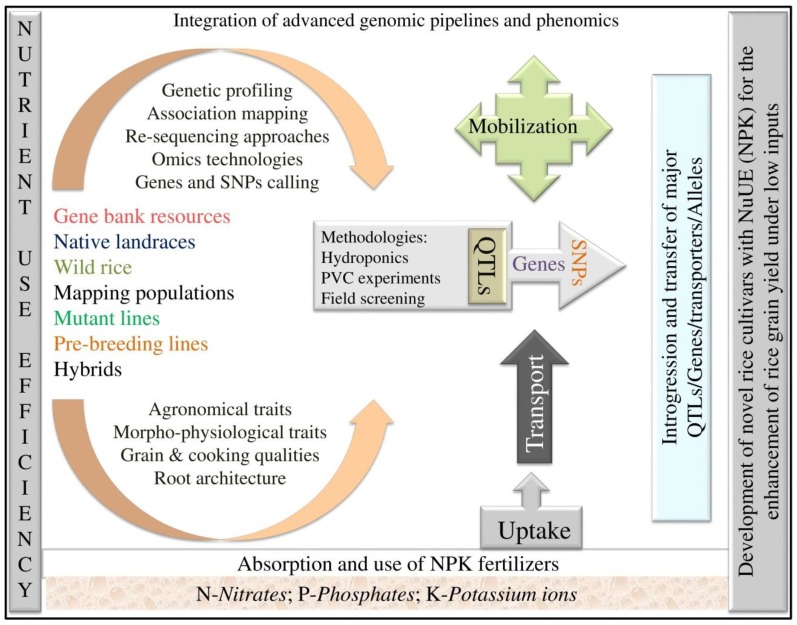
Integrated breeding and genomic approaches for improvement of rice cultivars superior in nutrient use efficiency (NuUE).

Understanding the genetic basis of agronomic, physiological and morphological traits in rice is critical for developing new and improved rice varieties. Rice breeders can use this information to select parental lines for hybridization and screen segregating populations (Figure 1). Recently, researchers have been gaining access to the enormous online wealth of genomic and plant breeding resources, including high-quality genome sequences [34,35,36], dense SNP maps [37,38,39], extensive germplasm collections and public databases of genomic information [35,36,39,40,41]. In this review, we have attempted to gather all the necessary information on QTLs related to N, P and K for the benefit of breeders involved in developing rice varieties with NuUE for sustainable agriculture.

## 2. Screening Protocols and Breeding Efforts for Traits Related to Nutrient Use Efficiency 

The literature is replete with NuUE screening protocols, especially for varieties, and very few are available for the systematic breeding of varieties with NuUE. Most of these NuUE studies use minus plots for different nutrients under study [42]. Research plots in institutions practice using omission or minus plots for any given target nutrient under study. Furthermore, researchers have always used natural sites with nutrient deficiencies for screening for any given nutrient such as the Pangil and Tiaong locations in the Philippines for P and Zn_ deficiency conditions, respectively.

### 2.1. Phosphorus

Deficiency of phosphorus is widespread in tropical and temperate acid soils. Screening and breeding for low phosphorus-tolerant (LPT) genotypes are some of the primary criteria for improving the use efficiency of P fertilizers. Worldwide, one-third of cultivable lands lack P in the soil to meet the requirement for ideal plant growth and development [43]. To avoid these stressful conditions, P is applied widely as an artificial fertilizer for improving grain yield for the burgeoning global population. The inconsistent use of fertilizers severely reduces income, and extreme conditions may cause environmental pollution [44]. Therefore, to overcome this crisis, the identification and improvement of P-efficient rice genotypes adapted to low-P soils would be a favorable solution for the enhancement of grain yield [45]. Developing P-efficient genotypes started with breeders involved in developing upland rice genotypes in an inadvertent manner. On the other hand, the mega-variety of India Swarna is a widely adaptable and popular variety among farmers perhaps because among its necessary traits is P responsiveness, as it possesses the *Pup1* QTL. Therefore, breeders should give more emphasis to developing lines tolerant of P_ deficiency with high grain yield. Krishnamurthy et al. [46] identified six LPT genotypes as Rasi, IET5854, IET14554, PRH122, IET15328 and IET17467, based on grain yield in field experiments at the Directorate of Rice Research in Hyderabad, India. Fageria et al. [47] reported seven lines (CAN 5164, CAN 4097, CAN 5170, IR3646-8-1-2, CAN 4137, A8-391 and IAC-47) at the National Rice and Bean Research Center of Embrapa in Brazil. In 2015, Saito et al. [48] found two varieties (Mudgo and DJ123) based on aboveground biomass at two locations. The development of such genotypes from diverse rice collections and mapping populations, along with cautious screening methodologies, is essential at the laboratory level to reduce the necessity for large-scale field evaluations. Several researchers used hydroponic nutrient solution and field experiments with different doses of P fertilizer to characterize rice varieties. This identified promising traits involved in tolerance of low P [48,49,50,51,52,53,54,55,56,57,58].

For grain yield and response to a graded level of applied phosphorus in low soil fertility conditions, Krishnamurthy et al. [50]) evaluated 28 pre-release promising rice varieties and hybrids at the Directorate of Rice Research farm in Hyderabad. They followed the protocol of 0–60 kg P_2_O_5_ ha^−1^ (i.e., 0, 10, 20, 30 40, 50 and 60 kg P_2_O_5_ ha^−1^) for the P application rate. Among the 28 rice varieties, four distinct patterns were identified in response to grain yield. Eight rice varieties at 0–10 kg P_2_O_5_ ha^−1^ and six varieties at 20–30 kg P_2_O_5_ ha^−1^ exhibited higher grain yield, while five varieties recorded higher grain yield in responses at higher P rates of 50–60 kg P_2_O_5_ ha^−1^. Out of the 28 varieties, three lines (IET 17190, Sumati and Rajavadlu) did not show any significant change in grain yield at 0–10 or 50–60 kg P_2_O_5_ ha^−1^, indicating the existence of genetic variability for P-use efficiency. Chin et al. [58] suggested a soil-based screening method as the most favorable approach for identifying genotypes with tolerance of P_ deficiency. Aluwihare et al. [53] experimented with Ultisol soils, without any application of fertilizer for four decades at Rice Research and Development Institute (RRDI), Sri Lanka, and this also confirmed the absence of P [58]. At P0 and P30 (30 mg/kg P_2_O_5_) conditions, during early vegetative, late vegetative and flowering stages, plant height (PH), number of tillers (NT), SDW (shoot dry weight), SPC (shoot P concentration), SPU (shoot P uptake) and PUE (P use efficiency) were found to be the major indicators for P_ deficiency tolerance (PDT). Among the total genotypes, 13 were considered as highly tolerant, 13 as moderate and 4 as sensitive to P_ deficiency based on SDW and P use efficiency under P0 conditions. Cancellier et al. [59] and Fageria et al. [60] elucidated that plant height is a vital morphological trait for PDT screening as it significantly correlates with dry weight and yield. Panigrahy et al. [61] identified four low P-tolerant and four susceptible mutants by screening 300-ethane methane sulfonate (EMS)-induced (Nagina 22 [N22]) mutants under low-P field conditions.

However, experimentations at the gene expression level were carried out in controlled test tube, Petri plate or potted conditions with different rates of nutrients, which often included the zero condition (control) for less than a month’s duration [13,62,63]. Li et al. [64] carried out expression profile studies using a DNA chip by subjecting rice at 6, 24 and 72 h under low-P stress and compared to a control treatment under normal P conditions. The study showed that genes directly involved in phosphorus absorption and use did not change significantly in transcription in rice shoots, relating to the inadequate low-P treatment. At 72 h under low phosphorus limitation, rice shoots did not develop severe phosphorus stress [65].

Specific genotypes known for their susceptibility to nutrient deficiency stress are useful for selection purposes, especially for different target nutrients. P_ deficiency tolerance was identified in a rice population derived from a cross between P-inefficient *japonica* cultivar “Nipponbare” and P-efficient *indica* landrace “Kasalath” [65].

On the other hand, several traits were studied to understand the phenotyping behavior of plants for precision screening and to progress in breeding activities. Root dry weight (RDW) is an important feature for evaluating the selection index for low-P tolerance in rice. Li et al. [49] reported that, at the seedling stage, dry weight had a significant genotypic variation (19.60%) in both standard and low-P conditions. TDW correlated with RRDW (relative root dry weight), RPH (relative plant height), RPUP (relative total P uptake), RSPA (relative shoot P accumulation), RPUE (relative P use efficiency) and RPC (relative P concentration) at *p* < 0.01. Several key morphological and physiological traits such as plant height, number of tillers, shoot root length, relative shoot and root dry weight and leaf age and root-attributed traits such as root diameter, root hair number and number of roots were used for screening and identifying tolerant genotypes under P_ deficiency conditions [61,66,67,68,69,70]. Increasing the productivity of grain yield under P_ deficiency conditions, increasing P taken up from the soil and improving the dry matter of internal use of P help to enhance the number of panicles and grain productivity [53,71]. Relative tiller dry weight (RTW), shoot dry weight and plant dry weight used as better screening criteria for identifying genotypes tolerant of low-P stress, especially RTW being sensitive, proved to be a reliable screening test. In recent days, image analysis has been becoming popular in high-throughput screening. Chen et al. [72] established an accurate, fast and operable method for diagnosing the crop nutrition status of NPK deficiencies in the color and shape of leaf parameters using a static scanning technology (SST) and hierarchical method in a pot experiment.

### 2.2. Nitrogen

Nitrogen fertilizer is an essential element for many aspects to improve grain yield, grain quality, flowering time and root development for extracting water and other nutrient elements from the soil [73,74]. On the other hand, the application of N is not uniform in all geographic regions of nations worldwide [75]. Several morphological and agronomic factors were found to influence the deficiency or high rates of N. Higher rates of N fertilizer consumption repeatedly led to environmental pollution and decreased nitrogen use efficiency (NUE) [76]. Therefore, the immediate focus should be to exploit the available variability in the use efficiency of rice cultivars through classical plant breeding methods and advanced biotechnological approaches to increase NUE in rice. Numerous research efforts have been conducted with different rates of N fertilizer in field experiments and hydroponic nutrient solution, and this was correlated with N use-efficient genotypes and higher grain yield (GY) parameters [77,78,79]. Chaturvedi [80] conducted a field experiment with different treatments of N fertilizer at the Agricultural Research Station in Chhattisgarh, India. Using an application of sulfur-containing nitrogenous fertilizer (Super Net) has significantly increased the grain yield and grain nitrogen content in hybrid rice variety Proagro 6207. Manzoor et al. [81] directed an experiment with nine different N rates (i.e., 0, 50, 75, 100, 125, 150, 175, 200 and 225 kg ha^−1^) at the Rice Research Institute in Lahore, Pakistan, with Super basmati. Interestingly, at 200 kg N ha^−1^ and above, yield-attributed traits declined, and higher grain yield, number of grains per panicle, 1000-grain weight, number of tillers and panicle length significantly improved at 175 kg N ha^−1^.

Likewise, Swamy et al. [82] evaluated ten rice genotypes under recommended rates of nitrogen (100 kg N ha^−1^) and deficient N as no external nitrogen (i.e., N0) in a treatment grown in field conditions at Indian Institute of Rice Research (IIRR), Hyderabad. They found that 14% of root length (RL) decreased significantly under N_ deficiency. Haque and Haque [83] detected higher grain yield (5.36 t ha^−1^) in 60 kg N ha^−1^, and the highest NUE (344.50 kg grain kg^−1^ N) was recorded for BU dhan 1 at six different N rates (0, 20, 40, 60, 80 and 100 kg N ha^−1^); they found an intermediate rate of N as economical and environment-friendly.

Employing a hydroponic experiment, Nguyen et al. [74] determined the effect of N supply in low and excess NH_4_NO_3_ concentration in Yoshida nutrient solution using three rice cultivars: IR64 (*Oryza sativa* ssp. *indica*), Azucena (*O. sativa* ssp. *japonica*) and TOG7105 (*O. glaberrima*). The rate of absorption of NUE (aNUE) and agronomic NUE (agNUE) decreased significantly, although at a gradual pace as the N supply increased, and physiological NUE (pNUE) declined progressively upon lowering the N supply.

To minimize N application and to use available N more efficiently, agronomic practices still need to be standardized. Nitrogen use efficiency is a complex trait and is associated with different components such as pNUE, aNUE, agNUE [77,78,84] and alteration in morpho-agronomic and physiological traits such as plant height, tiller number, grain yield, dry weight of shoots and roots, spikelet number, number of filled grains per panicle, 1000-grain weight, the leaf color chart (LCC) and chloroplasts [25,26,74,81,83,85,86,87,88,89,90,91] in rice. Alteration of the main traits was influenced by the response of N fertilizers, which may enhance the availability of N, which can lead to higher photo-assimilates and dry matter accumulation [80,92]. Therefore, considering the absorption, physiological and agronomic NUEs associated with morpho-agronomic traits will help to attain the balance between high grain yield and the eco-friendly nature of farm systems, which would be useful in developing crops with superior NUE.

### 2.3. Potassium

The availability of K in the soil is insufficient in developing countries, and it plays a significant role in crop grain yield and quality [93]. From 2012–2016, K fertilizer consumption globally increased from 28.6 Mt (K_2_O) to 33.2 Mt (K_2_O) [94]. Notably, East and South Asia are promising agricultural areas consuming 44.9% of the world K fertilizer, which is not adequate for improving grain yield under deficiency of K. The price of K fertilizers increased rapidly from 2003 (USD 165 per ton) to 2013 (USD 595 per ton) [94]. Therefore, the identification of K use efficiency (KUE) in rice is essential and needs to be used in developing genotypes with higher grain yield for K-deficient conditions. Dobermann et al. [95] mentioned that, as compared with other cereal crops, rice acquires 56–112 kg of K from soils in each harvest of yield of 4–8 t ha^−1^, and yearly K demand for irrigated rice would be 9–15 × 106 tons by 2025. In physiological aspects, K is involved in many functions related to regulating osmotic potential, transporting assimilates, root development for uptaking water and nutrients, reducing the frequency of diseases, drought tolerance and photosynthetic activity [96,97,98,99]. Under different rates of K fertilizer (0, 25, 50, 75 and 100 kg ha^−1^), Mehdi et al. [100] evaluated the response of rice cultivars in saline-sodic soil during 2005 and achieved the highest paddy yield (3.24 t ha^−1^) and straw yield (3.92 t ha^−1^) at 100 kg K_2_O ha^−1^. Similarly, Fageria et al. [101] elucidated lowland rice grain yield varying from 5.88–6.24 t ha^−1^ with an application of 125 kg ha^−1^ in different years. Analysis of six upland rice genotypes evaluated in a greenhouse under natural soil of 200 mg K kg^–1^ revealed that K uptake in shoot and grain and the K use efficiency ratio (KUER) were significantly and positively associated with grain yield [101], whereas, compared with grain, K concentration and uptake were higher in shoots. Arif et al. [102] conducted a pot experiment with three genotypes in a rain-protected wire house at the University of Agriculture in Faisalabad using hydroponic nutrient solution with different K rates of 0, 30, 60, 90 and 120 kg ha^−1^, respectively. Among the three genotypes, IR6 (low KUE), Super basmati (medium KUE), genotype 99509 (high KUE), the highest thousand grain weight (TGW) (IR6), grain yield (g pot^−1^) (Super basmati, 99509), number of panicles and tillers per pot (Super basmati) were recorded at optimum rates of 60 kg ha^−1^. Earlier reports revealed that a higher rate of K influences increases in yield-attributed traits [103,104,105,106]. The increase in yield with an optimum rate of K plays a crucial role in increased N use and increasing chlorophyll synthesis and translocation of assimilates to reproductive parts [107]. Recently, Islam et al. [108] compared the application of K fertilizer between 40 and 80 kg ha^−1^ in a randomized complete block design. The significant (*p* < 0.05) increases in grain and straw yield in the treatment with K application rates of 40 and 80 kg ha^−1^ were 54% and 68% in the dry season and 39% and 45% in the wet season from 2003–2010 in field experiments at the Bangladesh Rice Research Institute farm. Hence, improving uptake, transport and translocation of K efficiency in shoots and rice grain is possible for identifying superior genotypes to further enhance grain yield by proper management practices.

## 3. Identification and Use of QTLs Related to Nutrient Use Efficiency

Developing rice varieties with multiple tolerance is possible provided large-effect QTLs/genes are available and exploited with innovative molecular breeding approaches. The number of reported QTLs is unwaveringly increasing day by day, but still, very few are applied in breeding programs. Obtaining more data that validate QTLs/genes in different genetic backgrounds and environments is a prerequisite for their large-scale application. In rice, there is an attempt to bring a few large-effect QTLs that confer tolerance of submergence, drought, salinity and P deficiency together through molecular marker-assisted breeding. *Pup1* is the best model for exploiting the NuUE QTLs currently being used, for which molecular markers are now available and evaluated in different genetic backgrounds under field conditions [5].

### 3.1. QTLs Related to Nitrogen Use Efficiency

Among the essential nutrient elements, nitrogen is the most important one for rice growth in natural ecosystems. The green revolution, which was a breakthrough in agricultural production to secure human nutrition in the past century, depended mainly on fertilizer application and high-yielding modern varieties [109,110,111,112,113]. In this context, nitrogen use-efficient crop varieties are of great concern. Further, genes and QTLs related to agronomy for NUE are presented in Table 1 and Table 2. Deeper understanding of the molecular basis of NUE would enable us to provide valuable information for crop improvement through biotechnological approaches. Recent advances in genomics and proteomics approaches such as subtractive hybridization, differential display and microarray techniques are transforming our approach to identify the candidate genes that play a crucial role in the regulation of NUE [4,7,114,115,116,117,118]. In addition, marker-trait association for NUE through quantitative real-time polymerase chain reaction (RT-PCR) technology is being used [119,120,121]. The identification of potential candidate genes/proteins will serve as biomarkers in the regulation of NUE for screening genotypes for their nitrogen responsiveness. This will help to optimize nitrogen inputs in agriculture.

The modern rice varieties were all selected earlier for higher N uptake to obtain maximum grain yields. Conversely, the biggest problem with the increased N supply often leads to a decrease in N use efficiency. This is mainly due to high N uptake before flowering, but is also due to low N uptake during the reproductive growth phase and incomplete N translocation from vegetative plant parts to the grains [15,178]. Sustainable agriculture requires developing crop varieties with high yield potential and less dependency on heavy applications of N and P fertilizer. Similar to P, N has no systematic breeding program and screening protocol. The genotypes were screened either in nutrient minus fields or under solution culture.

In recent years, heavy nitrogen fertilization during panicle development has been popular in China to improve population dynamics and increase grain yield [179]. Panicle fertilization was adopted to increase grain yield and N recovery efficiency at IRRI [180]. Nitrogen use efficiency positively correlates with photosynthetic characteristics. The measures for promoting photosynthetic function and delaying senescence of leaves may indirectly enhance N absorption and use of rice and ultimately increase NUE. Some research efforts had been devoted to developing genotypes that use N more efficiently. This highly complicated objective requires an in-depth understanding of the genetic basis of N assimilation and N use at different developmental stages. The QTLs underlying related traits toward the late developmental stage in rice at two different nitrogen rates were investigated using a population of chromosome segment substitution lines (CSSLs) derived from a cross between Teqing and Lemont. A total of 31 QTLs referencing five traits, especially plant height, panicle number per plant, chlorophyll content, shoot dry weight and grain yield per plant, were detected. Under the normal nitrogen (150 kg/h^−1^ N fertilizer) rate, three QTLs were identified for each trait, and the under low nitrogen (0N) rate, five, four, five and two QTLs were detected for plant height, panicle number per plant, chlorophyll content and shoot dry weight, respectively. Most of the QTLs were located on chromosomes 2, 3, 7, 11 and 12 [166].

Based on the use of two N supply levels, 5 mg N L^−1^ for low N and 40 mg N L^−1^ [167] for high N, QTLs for plant height in rice were mapped onto the Restriction Fragment Length Polymorphism (RFLP) linkage map of a doubled-haploid population derived from a cross between IR64 and Azucena. Two QTLs, one on chromosome 1 and the other on chromosome 8, were detected at high N levels (40 mg N L^−1^) in soil-based nutrient solution culture experiments. Furthermore, a total of eight QTLs were identified at low N level and located on chromosomes 1, 2, 3, 4, 5 and 6, whereas the QTL flanked by molecular markers RZ730 and RZ801 on chromosome 1 was identified in all experimental conditions. The hypothesis suggests that the genotype showing higher N efficiency under low N level may carry the gene(s) for higher N efficiency. This study demonstrated that the effects of low N stress on plant height lessened. In the present study, the female parent IR64 was found to have a relatively higher N efficiency than the male parent Azucena under low N levels due to its lesser decline in plant height than Azucena. Furthermore, some of the QTLs associated with plant height were detected only at low N levels and might have some relationship with N efficiency [162]. QTL analysis was related to N and P tolerance traits such as root length at the seedling stage, productive panicles, seed setting ratio and yield. A few QTLs out of these were found to be located on similar chromosomal sections that showed the genes associated with the N or P metabolism pathway [181,182]. QTLs for rice panicle number and grain yield were detected under low nitrogen (N0) and low phosphorus (P0) conditions and helped to analyze the genetic basis of tolerance of soil nutrient deficiency. A total of 125 CSSLs with relatively few introgression segments were derived from *japonica* cultivar Nipponbare within the genetic background of *indica* cultivar 93–11. These were screened using an augmented design in field experiments with regular fertilization (NF), low nitrogen (N0) and low phosphorus (P0) treatments. Grain yield and panicle number per plant were measured for each CSSL, and their relative values based on regular fertilization treatment considered as the measurement for tolerance of the nutrient deficiency. Both regular fertilization and low phosphorus treatments showed adverse effects on grain yield and panicle number. The different responses observed among the CSSLs refer to the deficiency of nitrogen or phosphorus. The relative traits had a significantly negative correlation with the traits in the regular fertilizer treatment. Cultivar 93–11 showed higher tolerance of low-nutrient stresses than Nipponbare. The negative allelic effects of 38 QTLs were contributed by Nipponbare under nitrogen and phosphorus deficiency stresses. Out of these, 26 QTLs were responsible for yield and panicle number, and the remaining 12 QTLs specified the relative traits. Five QTLs were identified in common under both stresses. Moreover, 81% of the QTLs were specifically detected only in low nitrogen (N0) or phosphorus (P0) conditions. These different QTLs suggest that the response to limiting nitrogen and phosphorus conditions was regulated by various sets of genes in rice [168].

The application of N fertilizer is of particular importance for cultivating high-yielding rice. However, heavy nitrogen fertilizer uses with high loss of nitrogen in rice-growing areas have led to low N recovery rates and environmental pollution. Grain yields are used as an indicator of NUE since it is difficult to evaluate the amount of plant-available N from the soil or any source of N inputs, including fertilizer application and N fixation [183]. Genotypes with high NUE are those cultivars that produce high grain yields with the application of N, while those that do not yield well are genotypes with low NUE. Cultivars with high NUE have the ability to take up N and efficiently use it to produce grains [184]. The relative weight of root, shoot and plant under two different N treatments could reveal the cultivars showing tolerance of low N stress. The QTLs identified for relative performance were distinctive from those for root, shoot and plant weight detected under the two N treatment conditions [182].

The study of Tong et al. [174] revealed a correlation with path analysis indicating that spikelet fertility percentage had the most significant contribution to grain yield per plant at the 300-and 150-kg urea ha^−1^ rates, but filled grains per panicle contributed a strong positive relationship with grain yield per plant at the N0 level. Six of 15 QTLs identified with main effects were detected for each trait except SFP. Clusters of main-effect QTLs associated with several key traits were observed on chromosomes 1, 2, 3, 5, 7 and 10, respectively. The main-effect QTLs (*qGYPP-4b* and *qGNPP-12*) were identified at the N0 rate only, which explained 10.9% and 10.2% of the total phenotypic variation explained (PVE). The identification of genomic regions associated with yield and its components at different nitrogen rates will be useful in marker-assisted selection for improving the NUE of rice. The NUE-related trait in rice is so complex that different results were obtained in previous publications because of various experimental conditions, methods and materials. The main-effect QTL (M-QTL), epistatic QTL (E-QTL) and QTL × environment (Q × E) interactions of six traits were investigated using a fully-saturated simple sequence repeat (SSR) linkage map. Obara et al. [185] found a QTL region associated with panicle number and panicle weight on chromosome 2 that contains a regulator gene (*GS1*) for glutamine synthetase activity. The selected rice plants based on this QTL region showed superiority in tillering ability, panicle number and total panicle weight under low N rates.

Several researchers identified main-effect QTLs on chromosome 3 [171], chromosome 6 [186] and chromosomes 2 and 9 [170] by using doubled haploids and Recombinant Inbred Lines (DHs and RILs) populations.

Among these QTLs, one QTL was identified as being associated with the number of grains per panicle under low N rate, and it was located in a similar region to the *Pup1* locus on chromosome 12, thus encouraging the use of *Pup1* materials for testing low-N tolerance [5]. Recently, in a hydroponic experiment with CSSLs, Zhou et al. [118] identified a total 23 QTLs, with seven QTLs for N uptake (NUP) located on different chromosomes (2, 3, 6, 8, 10 and 11), with phenotypic variation (PV) ranging from 3.16–13.99%. Six QTLs for N use efficiency were located on chromosomes 2, 4, 6 and 10 and had explained PV ranging from 3.76–12.34%, respectively. The remaining 10 QTLs were responding to grain yield (GY) and biomass yield (BY). With the results of correlation analysis, Zhou et al. [118] suggested that both NUP and NUE had large effects on grain yield. Previous reports of Dong et al. [187,188] showed the NUP trait more closely associated with grain yield than NUE. NUE and NUP trait-linked QTLs are highly useful for improving grain yield under low-input conditions.

### 3.2. Phosphorus Use Efficiency and Related QTLs

Phosphorus is one of the essential macro-nutrients required for plant growth and development. Low availability of phosphorus in a variety of soils, especially in the tropics, often limits rice grain yields [189], along with the lack of available P sources locally in many countries. The higher importation and transportation costs of P fertilizers frequently prevent resource-poor farmers, especially in developing countries, from applying P to their deficient farmlands. Thus, developing rice cultivars with improved tolerance of P deficiency may therefore be a cost-effective solution to this problem. Rose and Wissuwa [45], optimistic that breeding for poor soil with high P uptake and high PUE needs to be developed and to maximize crop grain yield in such low-input systems, noticed that continuous cropping of poor soil is often related to poverty. It is also important to breed efficient crops. A combination of both P uptake and P internal nutrient efficiency is equally desirable for high-input systems, whereas it would facilitate a reduction in fertilizer rates without yield compensation. Dobermann and Fairhurst [190] reported in rice that P fertilizer use efficiency is only ~25%, which suggests considerable scope for improvement.

**Figure 2:**
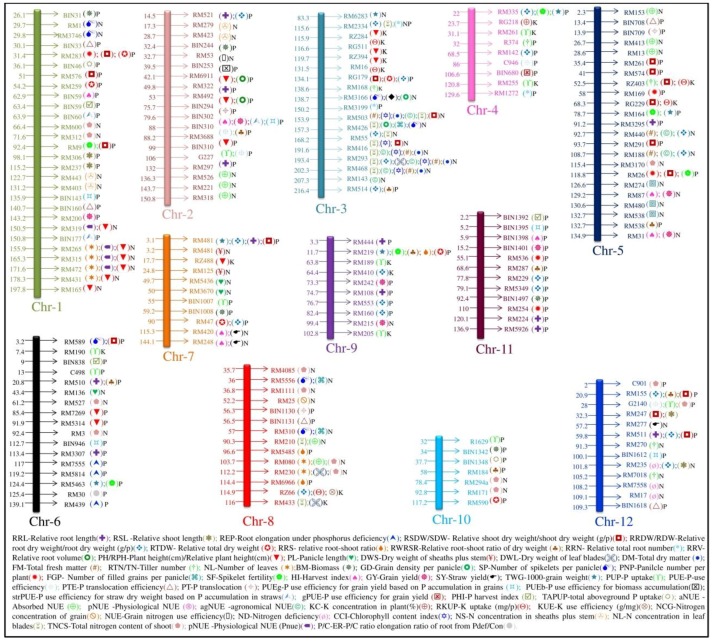
Diagram of 12 chromosomes with reported nutrient use efficiency (NuUE)-NPK QTLs linked to markers associated with the respective traits were identified through marker assisted selection (MAS) breeding approaches in a low-NPK environment using diverse mapping populations of rice.

Several researchers have identified genes and QTLs governing agronomic traits related to nutrient use efficiency, and these are shown in Table 1 and Table 2 and are represented in Figure 2 with the respective NPK QTLs located on 12 chromosomes associated with morpho-physiological traits under low-input conditions. The *Pup1* gene responsible for phosphorus uptake was identified and characterized by Chin et al. [57] (Table 1). Quantitative trait loci for P deficiency tolerance were identified in a rice population derived from a cross between P-inefficient *japonica* cultivar Nipponbare and P-efficient *indica* landrace Kasalath [65]. Tolerance of P deficiency was primarily caused by genotypic differences in P uptake; internal PUE had a negligible effect, and even phosphorus content changed slightly within 72 h in the shoots under low phosphorus stress, but phosphorus content decreased rapidly at 24 h in the roots [62].

Several studies were carried out to understand the genetics of tolerance of phosphorus deficiency in crops, and they identified several QTLs associated with it [54,55,56,57,58,59,60,61,62,63,64,65,66,154,156,191]. Su et al. [192] reported that 39 QTLs were associated with panicle number and weight of dry matter, chosen as the indices of P deficiency tolerance in wheat (*Triticum aestivum* L.).

The QTLs related to root traits, panicle number and seed set percentage were reported in rice [66,153,156]. Yield component traits such as panicle number and seed-setting percentage could be used as selection indices for P deficiency tolerance in rice [192]. However, only a few reports are available for the QTL mapping of grain yield and its components for P_ deficiency tolerance.

A significant QTL for P uptake was mapped to a 13.2-cM interval on the long arm of chromosome 12 flanked by markers C443-G2140. The position was estimated to be at 54.5-cM, a 3-cM distance from marker C443. Additional minor QTLs were found on chromosomes 2, 6 and 10 [155]. However, the first evidence supporting the presence of a significant QTL for P_ deficiency tolerance came from a study by Ni et al. [154].

A doubled-haploid population was derived from a cross between P_ deficiency-tolerant *japonica* rice IRAT109 and P deficiency-sensitive *japonica* rice Yuefu [193]. A total of 116 lines were evaluated for yield per plant and its component traits under P deficiency and normal conditions. There were significant differences in seed-setting percentage, panicle number per plant and yield per plant for the doubled haploid DH population between the two conditions, whereas there was no significant difference in 1000-grain weight and grain number per panicle. The results indicated that seed-setting percentage, panicle number per plant and yield per plant were easily influenced by P_ deficiency. Restricted fragment length polymorphism (RFLP) and simple sequence repeat (SSR) markers were used to cover 1535-cM of the rice genome to discover a total of 17 QTLs for plant yield and its components (1000-grain weight, seed-setting %, panicle number per plant, grain number per panicle) under P deficiency conditions. These QTLs explained from 2.65–20.78% of the phenotypic variance, with 12 QTLs showing higher than 10%. For 1000-grain weight, one QTL was detected, which had an logarithm of the odds LOD score of 5.13 and high contribution of PV (14.38%). Five QTLs were linked with seed-setting percentage, and three QTLs were linked with panicle number per plant [193]. Out of these five, three SP QTLs *(qSP2*, *qSP5* and *qSP11)* contributed more than 10%, and the three QTLs for panicle number per plant had high general contributions of more than 17%. Two QTLs (*qPN10* and *qPN12*) had an opposite additive effect. For grain number per panicle, four QTLs were detected, two of which (*qGN6* and *qGN7*) had high general contributions and positive effects. Four additive QTLs were found on chromosomes 2, 3, 6 and 7, which explained 4.77–13.55% of the phenotypic variance, for yield per plant. Three of them, *qYP3*, *qYP6* and *qYP7*, had high general contributions of more than 10% [194].

### 3.3. Potassium Use Efficiency and Related QTLs

Among the essential elements, potassium is necessary for plant growth. It is the activator of many enzymes in plants and the osmotic regulator of cell solute potential, and it plays a significant role in plant growth and metabolism. In rice, increased application of K fertilizer significantly improves grain and milling quality, such as increasing the percentages of brown rice, milled rice and head milled rice; reducing chalkiness; and enhancing grain protein content [194]. Fageria et al. [101] reported on K uptake and the use efficiency of upland rice under Brazilian conditions. They conducted a greenhouse experiment with the K rate as zero (natural soil level) and 200 mg K kg^–1^ of soil with the objective of evaluating the influence of K on grain yield, K uptake and their use efficiency, especially for six upland rice genotypes grown on a Brazilian Oxisol. Shoot dry weight and grain yield were significantly influenced by K rate and genotype treatments. The potassium concentration in the shoot was about six-fold greater than that of the grain, across two K rates and six genotypes. However, the K use efficiency ratio (KUER) was about 6.5-times higher in the grain than in the shoot, over two K rates and six genotypes. Potassium uptake in shoot and grain and KUER were significantly and positively associated with grain yield. Besides these, soil Ca, K, base saturation, acidity saturation, Ca saturation, K saturation, Ca/K ratio and Mg/K ratio showed a significant influence on the K application rate.

A greenhouse experiment was conducted at four levels of saline water irrigation (tap water and 2, 4 and 6 dS m^−1^ of salinity) and four different methods of K application (spraying with distilled water as the control, application of potassium on soil, potassium spraying and application of potassium on soil plus spraying). The purpose was to study the efficiency of potassium spraying and use in the soil and their effect on yield and its components under salinity stress. The results showed that grain yield, number of shoots, 100-seed weight, tiller number, dry root weight and K uptake in seeds and shoots decreased significantly with increasing salinity. The best method of K application was soil intake plus spraying [195]. In an investigation of a DH population consisting of 123 lines derived from *indica* variety IR64 and *japonica* variety Azucena under a hydroponic experiment, Wu et al. [177] identified three QTLs associated with shoot and root dry weight under K-deficient conditions. These same three QTLs were also influencing the effect on K content in the plant (KC), K uptake and K use efficiency. The QTLs individually had PVE ranging from 8–15% and were positioned on chromosomes 2, 3, 5 and 8 in K_ deficiency conditions.

## 4. Effect of Nutrient Use Efficiency across Medium- and Long-Duration Rice

Singh et al. [6] assessed the variability in grain yield and N use of 10 medium-duration (119 ± 4 days after seeding) and 10 long-duration (130 ± 4 DAS) genotypes. These genotypes showed varying rates of acquisition and use of soil and fertilizer N. Significant diversity within genotypes was found in grain yield and N uptake, efficiency and partitioning parameters (physiological N use efficiency, agronomic N use efficiency, apparent recovery, partial factor productivity (PFP) of applied N, N productivity index and N harvest index). The N use-efficient genotypes were IR54790-B-B-38, BG380-2 and BG90-2 (medium duration) and IR3932-182-2-3-3-2, IR54853-B-B-318 and IR29723-88-2-3-3 (long duration), producing high grain yields at both low and high rates of N, whereas inefficient genotypes produced low grain yields at low N rates, but responded well to N application. Increases in grain yields were highly correlated with N uptake. The grain yield-N uptake relationship for individual genotypes indicated significant differences in slope and the grain yield obtained with soil N (GY0). Significant differences in GY0 were due to genotypic variation in N uptake and efficiency of use. The N harvest index was related to both N uptake and use efficiency. The N productivity index, which integrated both GY0 and PFP of applied N, provided a better ranking of rice genotypes. The performance levels of efficient and inefficient genotypes over a range of soil and fertilizer N supply were consistent across three seasons of trials.

## 5. QTLs for Both Low Nitrogen and Phosphorus Stress

Eight QTLs explained panicle number per plant under the three treatments. Five of the QTLs were identified under the low-nitrogen treatment, and three were identified under the low-phosphorus treatment. The alleles from Nipponbare at all the QTLs_ had adverse effects on panicle number (decreasing it by 42.6–62.9%). No common QTLs were identified for panicle number under both low-N and low-P stresses. A total of 18 QTLs for yield per plant were detected in three treatments [175]. Located on chromosome 4, a QTL (*Qyd-4c*) was identified in all treatments with relatively higher phenotypic variance explained (58.2%, 55.2% and 88.1%) under normal, low-N and low-P conditions, respectively. The authors detected another four QTLs (*Qyd-3a*, *Qyd-4a*, *Qyd-7a* and *Qyd-10*) in two treatments. The rest of the 13 QTLs were identified in only low-nitrogen or low-phosphorus treatments. Regarding relative yield, two and three QTLs were identified in different N and P treatments, respectively, of which *Qryd-7a* was a common QTL, suggesting that the CSSL containing the *Qryd-7a* locus was sensitive to both N and P_ deficiency stresses [127,172]. QTL *Qyd-4a* was located in the same chromosomal region as the QTL for dry weight of seedling root [167]. The authors conjectured this substitution region to be associated with root response to nutrient stresses, probably containing genes for regulating nutrient absorption and consequently affecting yield per plant in rice. Root elongation gets hit by either N or P_ deficiency [126,167,172], resulting in various nutrition assimilation in plants. Several QTLs from this study correspond to known genes in the N or P metabolic pathway. For example, *Qyd-2b* for N_ deficiency tolerance was located near the gene encoding cytosolic glutamine synthetase (*GS1*), and *Qyd-3b* and *Qpn*-3 were nearby the genes for glutamate dehydrogenase *(GDH2)* [182]. Furthermore, *Qyd-12* was detected only under low-P conditions, and it co-localized with a significant QTL (*Pup1*) on chromosome 12, which was involved in P absorption [154]. These results indicate that the QTLs specifically detected under single N or P_ deficiency conditions may be involved in different pathways of N and P metabolism. Their tightly linked markers have breeding potential in pyramiding elite QTLs for N and P use efficiency.

Tolerance of low nitrogen stress conditions is a highly desired characteristic for sustainable crop production. The genetic components associated with low N tolerance in rice at the seedling stage, including main QTL effects, epistatic QTL effects and QTL by environment interactions, using a population of 239 RILs derived from a cross between popular Zhenshan 97 and Minghui 63, were studied [182] in solution culture. Root, shoot and plant weight over two N treatments were measured and the relative weight of the two treatments for each trait considered as measurements for low-N tolerance. Four to eight QTLs with main effects were detected for each of the nine traits. Very few QTLs were detected in both low and normal nitrogen conditions, and interestingly, most of the QTLs for the relative measurements were distinct from those for traits under the two nitrogen treatments, indicating very little commonality in the genetic basis of the traits and their relative performance under low and normal nitrogen conditions. In rice, some agronomic traits involving effective tiller number, spikelet fertility percentage and grain yield were studied under low nitrogen stress [166,170,185,196]. Two main-effect QTLs with large contribution rates were detected at the N0 rate. One of them affecting grain number per plant was detected at the interval RM117-RM101 on chromosome 12, accounting for 10.2% of the total phenotypic variance. There was no significant interaction between this M-QTL and environmental factors. This QTL is from the same region as a QTL (*Pup1*) related to phosphorus uptake [156]. Zhao et al. [33] reported that single segment substitution lines (SSSLs) each having a single chromosome segment derived from a donor under the same genetic background as the recipient parent were developed in rice by advanced backcrossing and genome-assisted selection. The QTLs for 22 essential traits were detected in rice with 32 SSSLs by a randomized block design in two to four cropping seasons. However, the QTLs controlling grain weight, grain length, the ratio of grain length to width and heading date were relatively stable. Fifty-nine QTLs were detected and distributed on chromosomes 1, 2, 3, 4, 6, 7, 8, 10 and 11, of which 18 were detected more than twice. Only 30.5% of the QTLs were repeatedly identified across different cropping seasons. Mostly the QTLs governing important agronomic traits showed small additive effects and instability. The stable QTLs usually had larger additive effects and were less affected by environment. With recent successful achievements in the Green Super Rice (GSR) project, efforts were made for highly adaptive rice cultivars with higher grain yield under low-input conditions [13,196,197,198,199,200,201]. About a 10% yield increase was obtained in elite GSR rice cultivars as compared with the local check variety NSIC Rc222 under multiple abiotic stress tolerance and low-input conditions, without compromising grain yield and quality [200]. Further progress in the genetic regulation of NuUE of GSR cultivars may provide valuable materials to understand the molecular and physiological pathways for the improvement of yield and grain quality under low-input conditions.

## 6. Agronomic Efficiency and Partial Factor Productivity QTLs

There is a significant increase in grain yield for each kg of fertilizer applied, termed agronomic efficiency (AE). Efficient fertilizer use is defined as maximum returns per unit of fertilizer applied [202]. According to Yadav [202], PFP and AE are useful measures of NUE, as they provide a basis for an integrative index that quantifies total economic output relative to the use of all nutrient resources in the system. Cassman et al. [203] defined PFP and AE to increase by increasing the amount, uptake and use of available nutrients and further by increasing the efficiency of applied nutrients that are taken up by the crop and used to produce grain.

Several researchers have studied AE and PFP in rice and other cereal crops. Dobermann [204] reported cereal crops in terms of AE of 10–30 kg grain kg^−1^ N, where >30 kg grain kg^−1^ is found in a well-managed system or at a low rate of N use or low soil N supply and for PFP 40–70 kg grain kg^−1^ N, with >70 kg^−1^ at low rates of N or in a well-managed efficient system. Wen-xia et al. [205] reported AE in two kinds of rice, one being Jinzao, with AE ranging from 8.02–20.14 kg grain kg^−1^ N, and the second one being Shanyou63, with an AE range of 3.4–18.37 kg grain kg^−1^ N absorbed. Yoshida [206]) estimated AE to be 15–25 kg grain kg^−1^ N, and Cassman et al. [203] reported AE at 15–20 kg grain kg^−1^ N in the dry season in farmers’ fields in the Philippines.

Amanullah et al. [207] declared that in maize, PFP for applied N was 36.62 kg grain kg^−1^ N and AE for applied N was 22.49 kg grain kg^−1^ N, using DAP and SSP in the field for the AE of two fertilizer applications, resulting in 13.01 and 13.71 kg grain kg^−1^ P, and PFP resulting in 63.58 and 61.92 kg grain kg^−1^ P. Rao [208] reported AE for applied K in hybrid cotton to be 8.8 kg grain kg^−1^ K, where the application rate of the fertilizer is NPK at 200-150-100 kg ha^−1^, and for non-hybrid cotton, 5.9 kg grain kg^−1^ K at the same rate of fertilizer application. In hybrid rice, AE for applied P was 5.2 kg grain kg^−1^ P and 11.8 kg grain kg^−1^ K with a fertilizer application rate of NPK of 200-75-200 and 200-150-200 kg ha^−1^, respectively. The AE for applied P in non-hybrid rice was 2.3 kg grain kg^−1^ P and 4.7 kg grain kg^−1^ P, where the fertilizer rate was the same. Rao [208] in another study showed that only the application of P (N and K as blanket doses) gave AE for non-hybrid rice of 4.2–15.6 kg grain kg^−1^ P and 5.9–11.4 kg grain kg^−1^ P, where the P application rate was 75 and 150 kg ha^−1^ and plant spacing was 12.5 × 10 cm and 10 × 10 cm, respectively.

The application of a unit of fertilizer is economical if the increase in crop yield due to the quantity of fertilizer added is higher than the cost of the fertilizer used. However, if a unit of fertilizer does not increase the grain yield enough to pay for its cost, then its application will not be considered economical and will not be profitable even after a constant increase in grain yield [209]. The application of essential plant nutrients in optimum split dosages and proportion, dispensed to plants in an appropriate method and timing, is the key to increased and sustained crop production.

## 7. Conclusions

Improving global rice yield productivity under low-input conditions is the main challenge for plant breeders and molecular biologists to develop/improve appropriate rice cultivars. Improving NuUE (nutrient use efficiency) is a key component from an agronomic, economic and environmental viewpoint. Despite the highly complex nature of NuUE in rice, recent trends in molecular marker-assisted selection and advanced biotechnological tools can accelerate the dissecting of the polygenic nature of complex traits. Apart from several breeding and agronomic strategies, balanced N, P and K nutrient elements are required to maintain soil fertility, uptake and transportation from soil to grain to produce higher grain yield with nutrient quality traits. The combined genomic and phenomic studies are valuable to distinguish the QTL and gene responses to NPK acquisition and transportation identified, and very few of them are strongly used with the target trait of interest in plant breeding programs. So far, plenty of QTLs have been identified in diverse genetic backgrounds with significant PVE under different treatment doses of NPK. By using this QTL information, better NuUE genotypes can be developed suitable for resource-poor farmers. Further, by employing these rapid developments, an integrative SNP array with innovative techniques such as Next-generation sequencing (NGS) and Genotyping by sequencing (GBS )technologies, high-density and SNP linkage maps and molecular breeding approaches are feasible solutions for identifying cultivars with superior NuUE by incorporating them into breeding cycles and understanding the molecular genetics and physiological mechanisms of N, P and K status in plants under different fertilizers or deficiency conditions. However, a combined holistic approach requires different aspects of work in the pipeline and omic technologies for its implementation in modern NuUE breeding programs.

## Figures and Tables

**Table 1:** Rice genes/QTLs governing key agronomic traits, the protein encoded, level of allele expression and their possible use in breeding programs.

S. No.	Traits	Name of QTL	Encoded Protein	Nature of Allele Suitable for Use in Breeding Programs	References
1	Grain number	Gn1a	Cytokinin oxidase	Low expression	[122]
2	Grain number and strong culm	dep1	PEBP-like domain protein	Loss of function	[123]
3	Grain number	WFP	OsSPL14	High expression	[124]
4	Grain number, low tiller number, and strong culm	Ipa	OsSPL14	High and ectopic expression	[125]
5	Grain size	gs3	Transmembrane protein	Loss of function	[126]
6	Grain size and filling	gw2	RING-type ubiquitin E3 ligase	Loss of function	[127]
7	Grain size	qSW5/GW5	Unknown	Loss of function	[128]
8	Grain filling	GIF1	Cell wall invertase	Restricted expression in the ovular vascular trace	[129]
9	Heading date	Hd1	CONSTANS-like protein	Loss-of-function allele leads to late heading	[130]
10	Heading date	Hd6	Subunit of protein kinase	Loss–of-function allele leads to early heading	[131]
11	Heading date	Hd3a	FT-like	Low expression leads to late heading	[132,133,134]
12	Heading date	Ehd1	B-type response regulator	Loss-of-function allele leads to late heading	[135]
13	Grain number, plant height and heading date	Ghd7	CCT domain protein	Functional allele	[136]
14	Days to heading	DTH8	CCT domain protein	Functional allele	[137]
15	Plant height	sd1	Gibberellin 20 oxidase	Loss of function	[138]
16	Lodging resistance	SCM2	F-box protein	High expression	[139]
17	Disease resistance	pi21	Proline-rich protein	Loss of function	[140]
18	Disease resistance	Pb1	CC-NBS-LRR protein	Functional allele	[141]
19	Salt tolerance	SKC1	HKT-type transporter	Gain of function	[142]
20	Cold tolerance	qLTG3-1	GRP and LTP domain	Functional allele	[143]
21	Submerge tolerance	Sub1A	ERF-related factor	Gain of function	[144]
22	Internode elongation under submergence conditions	SK2	ERF-related factor	Gain of function	[145]
23	Cadmium accumulation	OsHMA3	Putative heavy metal transporter	Functional allele	[146]
24	Seed shattering	sh4	Myb3 transcription factor	Loss of function	[147]
25	Seed shattering	qSH1	BEL1-like homeobox protein	Low expression in abscission layer between panicle and spikelet	[148]
26	Prostrate growth	PROG1	Zinc finger transcription factor	Loss of function	[149,150]
27	Disease resistance	RHBV	NS3 protein	Favorable gene or QTL alleles	[151]
28	Phosphorus uptake	Pup1	OsPupK46-2	High expression	[57]
29	Deep rooting	DRO1	Auxin signaling pathway	Functional allele	[152]

**Table 2:** Quantitative trait loci identified for traits related to nitrogen, phosphorus and potassium use efficiency in rice.

Entry	Phosphorus					
S. No.	Traits	Population	Cross	No. of QTLs		Reference
				M	E	
1	Phosphorus uptake, plant dry weight, tiller number; phosphorus use efficiency	NILs	*Nipponbare/Kasalath*	8	-	[65]
2	Relative tillering ability, relative shoot dry weight, relative root dry weight	RILs	*IR20/IR55178*	4	-	[153]
3	Phosphorus uptake, tiller number	NIL	*Nipponbare/Kasalath*	1 (Pup)	-	[154]
4	Root elongation, shoot dry weight, relative phosphorus content, relative Fe content	F_8_	*Gimbozu/Kasalath*	6	-	[155]
5	Relative root length, relative shoot length, relative shoot dry weight, relative root dry weight	BILs	*OM2395/AS996*	1	-	[156]
6	Root elongation under phosphorus deficiency	CSSLs	*Nipponbare/Kasalath CSSL29*	1	-	[157]
7	Plant height, maximum root length, root number, root volume, root fresh weight, root dry weight, shoot dry weight, total dry weight, root/shoot dry weight ratio	ILs	*Yuefa/IRAT109*	24	29	[63]
8	Relative root length, relative root dry weight, relative shoot dry weight, relative total dry weight, relative root-shoot ratio of dry weight	BC_2_F_4_	*Shuhui 527/Minghui 86*	48	-	[158]
9	Total aboveground biomass, harvest index, P use efficiency for grain yield based on P accumulation in grains, P harvest index, P translocation, P translocation efficiency, P total aboveground P uptake, P use efficiency for biomass accumulation, P use efficiency for grain yield, P use efficiency for straw dry weight based on P accumulation in straw	RILs	*Zhenshan 97/Minghui 63*	36	-	[159]
10	Root dry weight, relative shoot dry weight, relative total dry weight	DHs	*ZYQ8/JX17*	6	-	[160]
**Nitrogen**						
1	Plant height	DHs	*IR64/Azucena*	10	-	[161]
2	Rubisco, total leaf nitrogen, soluble protein content	BILs	*Nipponbare/Kasalath*	15	-	[162]
3	N uptake (NUP), grain yield, biomass yield, N use efficiency (NUE)	CSSLs	9311/Nipponbare	13		[118]
4	Toot system architecture, NDT, and morphological and physiological traits	CSSLs	Curinga/IRGC105491	13		[163]
5	Twelve physiological and agronomic traits	RILs	IR64/Azucena	63		[27]
6	Glutamine synthetase, glutamate synthase	BILs	*Nipponbare/Kasalath*	13	-	[164]
7	Glutamine synthetase, panicle number per plant, panicle weight	NILs	*Koshihikari/Kasalath*	1	-	[164]
8	Total grain nitrogen, total shoot nitrogen, nitrogen uptake, nitrogen use efficiency, nitrogen translocation efficiency	F_3_	*Basmati370/ASD16*	43	-	[165]
9	Root dry weight, shoot dry weight, biomass	RILs	*Zhenshan97/Minghui 63*	52	103	[166]
10	Plant height, panicle number per plant, chlorophyll content, shoot dry weight	CSSLs	*Teqing/Lemont*	31	-	[167]
11	Total grain number, total leaf nitrogen, total shoot nitrogen, nitrogen uptake, specific leaf nitrogen	RILs	*IR69093-4-3-2/IR72*	32	-	[168]
12	Root length, root thickness, root biomass, biomass, etc.	RILs	*Bala/Azucena*	17	-	[169]
13	Relative root dry weight, spikelet number per panicle, spikelet fertility, 1000-grain weight	ILs	*Shuhui 527 × Minghui 86*	48		[170]
14	Total grain number, total leaf nitrogen, total shoot nitrogen, physiological nitrogen-use efficiency, biomass	RILs	*Dasanbyeo/TR22183*	20	58	[170]
15	Total plant nitrogen, nitrogen-use efficiency	DHs	*IR64/Azucena*	16	-	[171]
16	Total plant nitrogen, nitrogen dry matter production efficiency, nitrogen grain production efficiency, total grain number	RIL	*Dasanbyeo/TR22183*	28	23	[172]
17	Grain yield per plant, biomass, harvest index, etc.	RILs	*IR64/INRC10192*	46	-	[173]
18	Plant height, root dry weight, shoot dry weight, chlorophyll content, root length, biomass	RILs	*R9308/Xieqingzao B*	7	-	[161]
19	Grain yield per plant, grain number per panicle	RILs	*Zhenshan 97/HR5*	19	11	[174]
20	Number of panicles per plant, number of spikelets per panicle, number of filled grains per panicle, grain density per panicle	RILs	*Xieqingzao B/Zhonghui 9308*	52	-	[175]
21	Nitrogen deficiency tolerance and nitrogen-use efficiency	RILs	*Zhenshan 97 and Minghui 63*	12		[176]
**Potassium**						
1	Plant height, tiller number, shoot and root oven-dry weight	DHs	IR64/Azucena.	4	-	[177]

M = main-effect QTLs; E = epistatic QTLs.
